# Food environment around schools and adolescent consumption of unhealthy foods in Addis Ababa, Ethiopia

**DOI:** 10.1111/mcn.13415

**Published:** 2023-03-31

**Authors:** Hanna Y. Berhane, Amare Worku Tadesse, Ramadhani Noor, Alemayehu Worku, Sachin Shinde, Wafaie Fawzi

**Affiliations:** ^1^ Nutrition and Behavioural Sciences Department Addis Continental Institute of Public Health Addis Ababa Ethiopia; ^2^ Department of Global Health and Population Harvard T. H. Chan School of Public Health Boston Massachusetts USA; ^3^ Department of Infectious Disease Epidemiology London School of Hygiene and Tropical Medicine London UK; ^4^ United Nations Children's Fund Ethiopia; ^5^ School of Public Health, College of Health Sciences Addis Ababa University Addis Ababa Ethiopia; ^6^ Epidemiology and Biostatistics Department Addis Continental Institute of Public Health Addis Ababa Ethiopia

**Keywords:** Addis Ababa, adolescents, Ethiopia, food environment

## Abstract

Adolescent diets may be influenced by the retail food environment around schools. However, international research to examine associations between the proximity of retail food outlets to schools and diet provides equivocal support for an association. This study aims to understand the school food environment and drivers for adolescents' consumption of unhealthy foods in Addis Ababa, Ethiopia. Mixed‐methods research was conducted, 1200 adolescents (10–14 years) from randomly selected government schools were surveyed, along with vendors within 5‐min' walk of the schools and focus group discussions (FGDs) with adolescent groups. Mixed‐effect logistic regression investigated the relationship between the number of vendors around the schools and the consumption of selected unhealthy foods. Thematic analysis was used to summarize findings from the FGDs. Consumption of sweets and sugar‐sweetened beverages (S‐SSB) and deep‐fried foods (DFF) at least once a week was reported by 78.6% and 54.3% of the adolescents, respectively. Although all schools were surrounded by food vendors selling DFF and S‐SSB, consumption was not associated with the number of vendors available around the school. However, adolescents' awareness and perception of healthy food, and their concerns about the safety of foods in the market, influenced their dietary choices and behaviours. Lack of financial resources to purchase food as desired also played a role in their selection of food and eating habits. Reported unhealthy food consumption is high among adolescents in Addis Ababa. Thus, further research is warranted to come up with school‐based interventions that promote access and healthy food choices among adolescents.

## Key points

- In‐school adolescents in urban Ethiopia frequently consume sweets and sugar‐sweetened beverages (S‐SSB) and deep‐fried foods (DFF).
- Adolescents' consumption of S‐SSB and DFF was not associated with the number of vendors selling those products around the school.
- Adolescents' awareness and perception of healthy and safe foods as well as their financial status influence their consumption.

## 1 INTRODUCTION

The prevalence of overweight and obesity among adolescents is on a steady rise (Di Cesare et al., 2019). Approximately, one in four children and adolescents globally are overweight or obese (Development initiatives, 2020). In Sub‐Saharan Africa, halting the prevalence of adolescent obesity is critical, especially in light of the already existing high burden of undernutrition (Onyango et al., 2019). The shift in diet from minimally processed and fresh foods to consumption of ultra‐processed foods is contributing to an increase in obesity as well as micronutrient deficiencies (Monteiro & Cannon, 2019; Monteiro et al., 2019; Zárate‐Ortiz et al., 2019), especially during adolescence. This period of life, identified as the second window of opportunity for growth, is a time of increased nutritional needs and a period where dietary habits and preferences are formed (Bush & Mates, 2017; Prentice et al., 2013). Therefore, access to a healthy diet during this period of life has the potential to address nutritional deficits from earlier ages; improve the health of adolescents now, and throughout their adult lives (WHO, 2018).

Globally, nearly nine out of 10 school‐age children are enroled in primary school (UNICEF, 2022), and consume at least one meal at or near the school they attend, making schools an ideal platform for reaching adolescents and shaping their dietary habits. The school food environment (SFE), which is a combination of the physical spaces, infrastructures as well as other policy and economic conditions in and around school that influence the availability, purchase and consumption of food, is a modifiable enabler of dietary choices among adolescents (FAO, 2021). As such, SFEs are critical for implementing comprehensive action for encouraging healthy diets and preventing diet‐related noncommunicable diseases among adolescents (World Health Organization, 2016). Recently, there has been considerable attention on how the SFE influences the dietary choices of adolescents. Studies have shown that retail food outlets in school vicinity are important sources of food for most students and influence their consumption; the presence of vendors and vending machines selling sweetened beverages in and near schools was found to have the potential to increase the consumption of sugar‐sweetened beverages (SSB; Davis & Carpenter, 2009; Rocha et al., 2021). Additionally, having a higher number of vendors selling unhealthy foods in proximity to schools was associated with lower dietary quality and an increase in body weight (Azeredo et al., 2016; Barrera et al., 2016).

As children age, they gain autonomy to exert more independent choices over their food options at school. Considering this, marketing efforts are increasingly targeting schools to create the demand for energy‐dense nutrient‐poor foods as opposed to healthy foods (Green et al., 2020; Kelly et al., 2008). Besides the actual availability of various foods to school‐going adolescents, the cost of food influences the type of outlets, and food preferred by adolescents (Colette et al., 2021; Trübswasser et al., 2021). There is a tendency that less healthy foods are less expensive (Billich et al., 2019), and thus are more consumed. In line with this, studies have shown that there is variability in the number of vendors selling healthy versus unhealthy food based on the neighbourhood‐level socioeconomic status and by school type—private versus public schools; unhealthy food availability was higher around public schools and in relatively low‐income neighbourhoods (Azeredo et al., 2016; Barrera et al., 2016; Díez et al., 2019). Furthermore, the presence of school meals (Patton et al., 2016), concerns about food safety and quality (Trübswasser et al., 2021) and the presence of promotion and marketing initiatives (Larson & Story, 2009; Monteiro et al., 2013) influence adolescents' consumption of food either positively or negatively.

The prevalence of obesity among adolescents in Ethiopia varies widely between studies, ranging from 3% to 18.2% (Gebrie et al., 2018). Ethiopia is undergoing rapid urbanization (World Bank Group, 2015), resulting in an explosion of food outlets and advertisements in urban areas, such as Addis Ababa (Melesse & Berg, 2020; Stuckler et al., 2012). Consequently, there has been a gradual shift in the diet as processed foods and food consumed away from home have become increasingly popular (Minten et al., 2018; Sheehy et al., 2019; Worku et al., 2017). The increasing cost of healthy foods (fruits and vegetables) (Bachewe et al., 2017) further contributes to the dietary shift. Although the rates of obesity are relatively low among Ethiopian adolescents at present with a pooled estimate of 2.4% (Gebrie et al., 2018), trends from other low‐middle‐income countries show that often these kinds of dietary changes are followed by an increase in obesity rates (Ford et al., 2017). A school environment that promotes healthy eating has a positive effect on adolescents' eating behaviours (Driessen et al., 2014; Rathi et al., 2016); nonetheless, most of the evidence comes from studies in high‐ and middle‐income countries, with limited research conducted in African countries (Carducci et al., 2021). Understanding the SFE and drivers for adolescents' consumption of unhealthy food in low‐income settings could serve as a basis for developing policies that could slow the nutrition transition.

This study examined the relationship between the availability of food vendors around schools and adolescents' consumption of unhealthy foods in Addis Ababa, Ethiopia. Additionally, we explored what influences adolescents' consumption of unhealthy foods.

## 2 METHODS

### 2.1 Study setting

The large majority (93%) of primary schools in Ethiopia are government‐owned (FMOE, 2020). In most government‐run primary schools, a national school feeding programme has been launched to improve school attendance and the diet diversity of children (FMOE, 2015). In Addis Ababa, the largest city in Ethiopia, school attendance is high, with nearly 92% of its population having attended at least some primary education (Central Statistical Agency (CSA) [Ethiopia] & ICF, 2016). Slightly more than half a million children are enroled in primary schools (grades 1–8) with a female to male ratio of 1:2 (FMOE, 2020).

### 2.2 Study design

This study used a mixed‐methods approach, including a quantitative survey with adolescents, vendors mapping and characterization, and focus group discussions (FGDs) with adolescents.

### 2.3 Quantitative approach

A cross‐sectional study was conducted in 20 government schools in Addis Ababa. A multi‐stage sampling approach was used: in the first stage, we randomly selected two schools from each subcity in Addis Ababa; then one section from grades 5–8 was selected in each school; finally, 15 students per section were selected randomly. The list of schools from the education bureau of the city and the school rosters were used as sampling frames, respectively. Furthermore, in each selected school, all vendors within a 5‐min walking radius around the school were included in the study totalling 343 vendors.

Interviews with the selected students and vendors were conducted using pretested structured questionnaires. The data collection tools were initially prepared in English and translated to Amharic for the interviews. Before conducting the fieldwork, all research assistants were trained on the study objectives, study tools, human subjects' protection, and interviewing skills. Survey tools were pretested and uploaded onto a tablet for data collection.

The student questionnaire included sections on demographics and socioeconomy, eating behaviours, dietary quality and food security. The dietary questionnaire asked the question ‘How often did you eat the following foods over the last week?’ To define categories of foods we adapted the food list from the Prime Diet Quality Score (Gicevic et al., 2018); group 1 components included healthy food items, and group 2 components comprise unhealthy foods. For this study, we focus on data pertaining to three food categories: SSB (e.g., soft drinks, energy and sports drinks; exclude sugar‐added fruit nectars, milk or cereal‐based sugary drinks, fruit syrups and juices), sweets and ice cream (e.g., candy, chocolate, cake, cookie, sugar cane and ice cream) and fried foods eaten away from home (e.g., fried food sold as street food, fast food and any of the fried foods sold for eating out). Consumption was defined as having consumed any fried food, sweets or SSB at least once a week; consumption was categorized (as never consumed vs. consumed at least once a week).

Other covariates considered in this study were parental education and household hunger. Parental education was assessed based on the adolescent's reported highest level of education received by their father/male guardian and mother/female guardian. The response options were recorded as none, primary, secondary, technical/vocational, university/college, don't know or don't have father/mother or male/female guardian; further, during analysis, we categorized the groups as those having none or lower than primary education and those having secondary education and above excluding don't know and don't have guardian options from our analysis. The household hunger was assessed using the USAID FANTA household hunger scale (Ballard et al., 2011). The household hunger scale uses a recall period of 30 days asking about the occurrence of an event and its frequency. The household hunger score was then computed ranging from 0 to 6 as per the guide (Ballard et al., 2011), and later grouped into two levels—households that experienced little or no hunger and households that experienced moderate or severe hunger.

The vendor questionnaire included information on the availability of specific food groups (fruits, snacks, beverages, dairy, deep‐fried foods [DFF] and processed foods). It asks the question: ‘Is this vendor selling food group (listing each of the above‐mentioned food groups) today?’ Response categories include Yes/No. The main exposure variable considered for this study is the number of vendors selling packed foods, snacks and DFF around schools, thus we used the count of each vendor selling the different products. Finally, we dichotomized SFEs using the mean as a cut‐off. For the vendor type, we had predetermined types, namely, Souq/kiosk (shop selling over a window and has a wide range of products from fresh to highly processed packed food), café and restaurants, tea house (informal structures often selling tea/coffee and fried street food), bakery, street vendors (mobile or permanently stationed vendors with informal structures selling different fried, packed or fresh fruits), fruit/juice shop (shops selling typically fruits/vegetables and also serve fresh juice), liquor stores, mini/supermarkets and other categories, which were developed based on the research teams contextual experience and refined after the pretest.

STATA 14 software was used for data analysis; frequencies and percentages were calculated to report descriptive statistics. A mixed‐effect logistic regression model was used for analysis; in the first model, we tested the association of consumption of unhealthy foods (fried foods, sweets and SSB) with having an SFE having vendors below/above the mean number of vendors selling those products. In the second model, we controlled for the adolescent's age and sex. Finally, we further adjusted for household wealth, hunger and paternal education. All three models account for the clustering effect by the school. The mean number of vendors selling fried foods and beverages was (4 ± SD 2) and (12 ± SD 6) vendors, respectively. Since vendors selling sweets were not measured, we used those vendors selling packed foods or snacks as a proxy to categorize the SFE that sold sweets (mean = 15 ± SD 6).

### 2.4 Qualitative approach

FGDs were conducted with mixed groups (three boys and three girls in each group) in four randomly selected schools out of the 20 initially selected for the adolescent survey. Students within the age group 10–14 were selected purposively; a school representative recommended the students based on their participation/engagement in school activities. Topics covered during the discussion included: being healthy and how adolescents perceive health, school policies, eating habits, access to food and preferences, including probes on reasons for selecting certain food over others.

FGDs were conducted using a discussion guide in Amharic by experienced research assistants who had a minimum of Master's level education. The discussion was conducted in a quiet space in the school outside of school hours upon getting parents/guardians' permission and assent. The duration of the FGDs ranged from 90 to 120 min. Informed written consent was obtained from the parents/guardians of the students 1 day before the discussion and verbal assent was taken from participants just before the beginning of the session. Besides a full audio recording, detailed notes were taken in each session. Recordings were transcribed and translated verbatim to English for analysis. The quality of the transcripts was verified by members of the research team by listening to the audio recording. Transcripts were open‐coded, and themes emerged inductively. The preliminary results were then presented and discussed among peers, coauthors and context experts to fine‐tune the results as well as identify alternative interpretations/meanings. The findings of these FGDs helped provide an alternative explanation as to what influences adolescent consumption of unhealthy food in our setting and offer a more balanced explanation to readers.

### 2.5 Ethical consideration

Ethical clearance was obtained from the Institutional Review Boards of Addis Continental Institute of Public Health (ACIPH) and the Harvard School of Public Health (HSPH). Additionally, support letters were obtained from Addis Ababa City Administration Education Bureau and subcity education offices. All school directors were approached upon receiving these support letters. Informed consent from the school directors, parents, adolescents (assent) and vendors was obtained before conducting the interviews/FGDs.

## 3 RESULTS

### 3.1 Quantitative results

#### 3.1.1 Study participant characteristics

This study enroled a total of 1200 adolescents. The mean age among the study participants is 12.6 ± 1.2 years. The majority were female (54.8%) and have not worked in the last 12 months (92.7%). Nearly half of the adolescents live with neither parent (49%). When we examined parental education, 51% of fathers and 33% of mothers have a high school education or above. When assessing the consumption of unhealthy foods in the past week, 54% and 79% of the adolescents had consumed fried foods and SSB at least once in the past week, respectively (Table 1).

**Table 1:** Description of adolescents in the selected government schools, Addis Ababa, Ethiopia (*n* = 1200)

Variable	Frequency	%
*Age (years)*		
10	54	4.5
11	188	15.7
12	294	24.5
13	344	28.7
14	320	26.7
*Sex*		
Male	543	45.25
Female	657	54.75
*Have you done work in the last 12 months?*		
No	1112	92.7
Yes	57	4.8
Don't know	31	2.6
*Currently, living with*		
Both parents	404	33.7
Mother	179	14.9
Father	30	2.5
Neither	587	48.9
*Father's education (n = 662)*		
Primary/no education	326	49.2
High school and above	336	50.8
*Mother's education (n = 973)*		
Primary/no education	655	67.3
High school and above	318	32.7
*Household hunger*		
Little or no	1157	96.4
Moderate/severe	43	3.6
*Consumption of fried food in the past week?*		
Never	548	45.7
Once/week	305	25.4
2–4 times/week	301	25.1
5–7 times/week	37	3.1
Once/day	5	0.4
Don't know	4	0.3
*Consumption of sweets and sugar‐sweetened beverages in the past week?*		
Never	257	21.4
Once/week	416	34.7
2–4 times/week	425	35.4
5–7 times/week	80	6.7
Once/day	19	1.6
Don't know	3	0.3

#### 3.1.2 Characteristics of vendors around school premises

A total of 343 vendors were identified in and around the 20 schools. Most of the vendors were located outside the school property (94.2%). Commonly available vendor types were Souq/kiosk (49.3%), followed by cafés and restaurants (14.3%). Snacks (67.4%), beverages (71.4%) and processed foods (50.7%) were the most available food items with the vendors located in and around the school, while fresh fruits were the least available (Table 2).

**Table 2:** Description of the vendors available around government schools in Addis Ababa, Ethiopia (*n* = 343)

Variable	Frequency	%
*Vendor location*		
Inside school	20	5.8
Outside property	323	94.2
*Vendor type*		
Souq/kiosk	169	49.3
Café and restaurants	49	14.3
Tea house	34	9.9
Bakery	29	8.5
Street vendor	18	5.3
Fruit/juice shop	14	4.1
Other	13	3.8
Liquor store	11	3.2
Mini/supermarket	6	1.8
*Vendors selling selected food items*		
Fresh fruits (yes)	36	10.5
Snacks (yes)	231	67.4
Beverages (yes)	245	71.4
Dairy (yes)	111	32.4
Deep‐fried foods (yes)	78	22.7
Processed foods (yes)	174	50.7

#### 3.1.3 Does the number of vendors available around school affect the consumption of unhealthy food?

When evaluating the association between the consumption of DFF with the number of vendors selling those products around the school, the results were not significant (adjusted odds ratio [AOR], 0.94; 95% confidence interval [CI], 0.54–1.63) even after controlling for potential confounders. Similarly, no significant association was observed between the consumption of sweets (AOR, 0.89; 95% CI, 0.60–1.24) and SSB (AOR, 0.86; 95% CI, 0.56–1.31) and the number of vendors around the school selling those products even after controlling for potential confounders (Table 3).

**Table 3:** Association between consumption of fried food by adolescents and the number of vendors available around schools

	Consumption of fried foods				
	Never consumed	Consumed at least 1/week	Unadjusted OR (95% CI)^a^	Adjusted OR (95% CI)^b^	Fully adjusted OR (95% CI)^c^
*SFE having*					
≤3 vendors selling DFF	231	309	Ref	Ref	Ref
4+ vendors selling DFF	317	343	0.81 (0.55–1.19)	0.81 (0.55–1.20)	0.94 (0.54–1.63)
*Consumption of sweets*					
≤14 vendors selling PF/S	230	430	Ref	Ref	Ref
15+ vendors selling PF/S	179	361	1.08 (0.81–1.45)	1.09 (0.81–1.46)	0.86 (0.60–1.24)
*Consumption of sugar‐sweetened beverages*					
≤11 vendors selling SSB	408	432	Ref	Ref	Ref
12+ vendors selling SSB	186	174	0.88 (0.66–1.19)	0.88 (0.65–1.18)	0.86 (0.56–1.31)

Abbreviations: CI, confidence interval; DFF, deep‐fried foods; OR, odds ratio; PF/S/Bev, packed food, snack and beverages; Ref, reference group; SFE, school food environment; SSB, sugar‐sweetened beverages.

^a^ Model 1 = Unadjusted.

^b^ Model 2 = Model 1 + adjusted for adolescent age and sex.

^c^ Model 3 = Model 2 + household wealth quintile, household hunger and mother's and father's education; clustering effect was controlled for all models.

#### 3.1.4 Qualitative results

A total of 24 adolescents participated in four FGDs (12 boys and 12 girls aged 10–14 years). Two major themes emerged that helped to better understand what influenced adolescents' diet and consumption: (1) awareness and perception of healthy and safe foods and (2) financial limitations.

*Theme 1—Awareness and perception of healthy and safe foods*

The participants had good knowledge about the importance of a nutritious diet. They were aware of the need to consume different fruits and vegetables to get sufficient vitamins to grow and prevent diseases.

I like fruits and vegetables because it is a source of vitamins and it helps me with body fitness. Additionally, it will increase my learning capacity. (S4B2)

I have received information from TV and books, about the importance of different vegetables and fruits … how it helps our health. For example, I have read fruits and vegetables prevent disease … eating those foods help me to have disease resistance. (S1G4)

In addition to their awareness of the importance of consuming healthy food, most have heard that some of the foods sold outside might be unsafe. Their concern towards the safety of the food that is prepared on the streets as well as those of packed food made them limit the purchase of those food items. Some students mentioned that they have heard information on television and other media that some of the packed foods sold on the streets are unsafe making them doubtful about consuming food sold on the streets. On the contrary, others mentioned that due to fear of food hygiene and safety concerns about the food prepared on the streets they opt for packed food, which was perceived to be safer.

Since it is packed, even though we can't say its clean, it is better than the pasty (deep‐fried street food. (S3F3)

There are foods that are out of date and forbidden to eat but sold on the market … they told us not to buy these foods from the market. They also told us to see the expiry date on the packaging … this was also told on the radio. (S2B1)

I won't buy anything … because in a recent TV show packed foods were shown to be not good for our health … including the plastic package. I also don't buy street food … because of the oil. (S3B6)

The oil used to fry them (referring to the fried foods) may last for a long time … a month or more … if I had money, I would buy packed foods. (S3G2)

*Theme 2—Financial limitations*

The adolescents indicated that although they would have liked to purchase food from outside, such as Chornake (fried dough), fries, donuts, biscuits and sweets, they often do not have the money to do so:

For me getting money … is a rare thing. I may get money from my grandparents or my father on occasions. (S1B3)

I get money occasionally, when I do, I buy either chips, biscuits, or Merenda (sweet drink). (S4G5)

Most students mentioned their families cannot afford to provide them with pocket money that covers anything beyond school necessities. Besides, if it was not for the school feeding programme, many children would not even have the opportunity to get a sufficient amount of food for the day let alone spend money for snacks. According to the participants, lack of financial resources restricted them from purchasing any food outside, be it healthy or unhealthy, despite the availability of a variety of food for sale both within and outside of the school compound. The participants repeatedly used the words ‘if I get some money’ and ‘when I get some money, I would opt for the cheap and sweet things’. When asked ‘do you often get the food you want to eat?’, they indicated that different kinds of food are available in and around school, however, the most important determinant as to whether they can consume it or not was their finance.

It usually depends on our financial ability. We may not find what we want even at home. (S3B3)

… inside our school compound … there is a tea shop. There you can find different food being sold like Beyeaynet (a local mixed dish served with Injera [traditional flatbread]), Shiro (legume‐based stew served with Injera). You can also find tea, coffee, and bread. Ice is also sold when we get out of the compound. I sometimes also see pasti (local name for a kind of biscuit that is deep‐fried) and other biscuits being sold. When you go out of the compound, you will see a doughnut shop. (S3G5)

I do not usually get money. But when my parents have money, they sometimes will give me … when that happens, I often buy chips … because it cheap and the vendor is near my home. (S2G2)

## 4 DISCUSSION

The results of the study showed that 78.6% and 54.3% of primary school students within the age group (10–14 years) consume sweets and sugar‐sweetened beverages (S‐SSB) and DFF at least once a week, respectively. There were a wide variety of vendors around the school premises, predominantly selling deep‐fried and packed foods. When testing the relationship between the number of food vendors around the school with consumption of S‐SSB and DFF, we found that consumption was not associated with the number of vendors selling those products around the school. Rather upon further exploration during FGD, we found that adolescent consumption of DFF and S‐SSB was limited due to lack of money. Even during their occasional purchase, some adolescents were worried about the safety of the foods available in the market.

More than half of the adolescents in our study were consuming S‐SSB and DFF at least once a week. This finding is consistent with findings from India and Brazil, which report increased consumption of energy‐dense snacks and beverages among adolescents (D'Avila & Kirsten, 2017; Rathi et al., 2017). In Ethiopia, although there are very limited studies focusing on the consumption of highly processed energy‐dense foods, little evidence available indicates there is a gradual shift. When assessing the diet transformation and trends in energy and nutrient supply in Ethiopia, studies found there is a slow increase in the consumption of processed and convenient foods, as well as in the use of sugar (Minten et al., 2018; Sheehy et al., 2019). In line with this, a recent review of processed food in Africa highlighted that over the past five decades there has been a consistent increase in the purchase and consumption of processed and ultra‐processed foods (Reardon et al., 2021). Considering that increased consumption of highly processed energy‐dense food has been associated with various health problems, including metabolic syndromes, obesity and other diet‐related noncommunicable diseases at different life stages (Durán‐Agüero et al., 2021; Nardocci et al., 2021; Tavares et al., 2012), it is essential to conduct more contextual studies to further understand the consumption patterns and come up with interventions that promote healthy eating choices.

One of the common drivers of the different forms of malnutrition is the food environment (Hawkes et al., 2020), and for this study, we focused on the SFE, which is presumed to be important for adolescents' diets. Studies from Brazil and Guatemala found that SFEs, particularly those having vendors selling unhealthy food, including SSB and salty fried snacks in the vicinity were correlated with increased consumption (Azeredo et al., 2016; Godin et al., 2017). Contrary to this, our findings show the availability of DFF as well as S‐SSB around the school were not associated with adolescents' consumption. In accord with our findings, studies from higher income countries, like, Canada and the United States, also have found that SFE‐level characteristics had a modest to negligible association with adolescent consumption of SSB, and point towards the importance of examining other factors, like, individual level, or other contextual factors, like, residential and home food environments (Godin et al., 2019; Haughton et al., 2018). Although the contexts may not be comparable, this could be one explanation for our results and point towards the need to further explore other individual, social, cultural and contextual factors that could affect adolescents' consumption of unhealthy food in Addis Ababa. Another potential explanation for differences in our results could be our sampling approach; we included only government school students. While this group of students represents the majority in Addis Ababa, where our study was carried out, adolescents attending public schools often represent the lowest‐socioeconomic groups of the population with little or no additional resources to spend on purchased food. They rely mainly on the school feeding programme for food, which is in a way protecting them from accessing purchasing unhealthy foods. The school feeding programme could thus serve as a window of opportunity for planning future interventions for obesity prevention in addition to its current objective, which is to curb the problem of undernutrition.

The quantitative model adjusting for wealth status did not find a significant association between the number of sellers and consumption; however, the qualitative results suggest that finance plays an important role. Adolescents' limited access to income/money was preventing them from purchasing unhealthy food. A school‐based study from Brazil found that students' socioeconomic status was associated with the consumption of unhealthy foods, with those with lower socioeconomic status consuming more unhealthy food (Azeredo et al., 2016). In our context, the relative price of unhealthy foods is also expensive, which would potentially explain the differences in results. Our results could potentially be different if we sampled from a private school that would have access to pocket money. Although not directly measuring the consumption of unhealthy food, a recent systematic review revealed that children and adolescents going to private schools and those from the highest income households were more than three times more likely to be overweight/obese compared with their counterparts (Gebrie et al., 2018). A study from Saudi Arabia also found that the density of fast‐food outlets was significantly higher around private schools compared with government schools, which could be indicative of demand around private schools (AlQurashi et al., 2021). In our study, despite the fact that limited access to money limited the consumption of unhealthy food, almost eight out of 10 adolescents also consumed S‐SSB. This contradictory result may be explained in part by the Ethiopian food culture, in which food is shared among friends and family. Therefore, one must consider that consumption could be a bite/small piece or even a sip of these foods and drinks that were reported as consumption by the adolescents. Thus, we need to have further studies to understand the food culture among adolescents and a more robust measure that includes the amount consumed.

Although financial access was a barrier to accessing unhealthy food, another recurring concern students raised was the safety of the food. Food safety is a rising concern in Africa due to the rapidly evolving food systems and their complexities (Global Food Safety Partnership, 2018); in Addis Ababa, studies have found that the residents are worried about poor food hygiene and preparation practices among food handlers as well as the substandard imported items (Berhane et al., 2018). Trübswasser et al. (2021) also found that adolescents opt for packaged food as they consider them to be relatively safer, as opposed to fresh food (fruits, vegetables as well as cooked food) sold on roadsides, which were considered unsafe and unhealthy. Enforcing food safety guidelines for food outlets could be a potential strategy to alleviate fear and promote the consumption of healthy food among adolescents.

The strength of this study is the use of mixed methods, to connect the results of the quantitative and qualitative to better understand whether the number of vendors within the vicinity of the school influences the consumption of unhealthy foods and further explore other influencing factors. The present study has limitations: First, it may not be possible to generalize this finding to other regions of Ethiopia as Addis Ababa is peculiar as it is the only region with entirely urban areas. It may however be plausible to cautiously relate this finding to urban centres in Ethiopia as well as other similar cities in Sub‐Saharan Africa considering cultural variations. Second, despite our best efforts, we cannot entirely rule out the effect of social desirability on our results; if the adolescents had considered consumption of the food we asked about as positive it is likely they may have inflated their consumption. Third, our assessment of the food environment captured only the availability of vendors and has not considered other dimensions of the SFE, like, nutritional quality and adequacy of school meals, which should be considered in future studies to paint the full picture of the SFE in Addis Ababa.

## 5 CONCLUSIONS

Reported unhealthy food consumption is high among adolescents in Addis Ababa. Without early interventions, these dietary shifts and contextual influencers might lead to an increase in overweight and obesity, putting Ethiopia under a double burden of malnutrition. The limited resources of the country will make it difficult to deal with the double burden of malnutrition; therefore, policies/plans for preventing undernutrition and decreasing overweight and obesity among adolescents need to be devised in advance.

## AUTHOR CONTRIBUTIONS

Hanna Y. Berhane, Amare Worku Tadesse, Ramadhani Noor, Alemayehu Worku, Sachin Shinde and Wafaie Fawzi designed the study. Amare Worku Tadesse supervised field operations and data collection. Hanna Y. Berhane and Alemayehu Worku carried out data analysis and interpretation. This paper was written by Hanna Y. Berhane with substantial input from all authors. All authors have reviewed and approved the submitted manuscript.

## CONFLICT OF INTEREST

The authors declare no conflict of interest.

## Data Availability

The data that support the findings of this study will be made available upon request pending application and approval by the study team.
